# The Effects of* Morus alba* and* Acacia catechu* on Quality of Life and Overall Function in Adults with Osteoarthritis of the Knee

**DOI:** 10.1155/2017/4893104

**Published:** 2017-09-11

**Authors:** Douglas S. Kalman, Susan J. Hewlings

**Affiliations:** ^1^QPS, Springfield, MO, USA; ^2^Central Michigan University, Mount Pleasant, MI, USA; ^3^Substantiation Sciences, Weston, FL, USA

## Abstract

The purpose of this study was to determine the effects of UP1306 on discomfort and function in adults with osteoarthritis of the knee. In a randomized, double-blinded, placebo-controlled, parallel design, 135 subjects received UP1306, a standardized, proprietary extract of* Morus alba* and* Acacia catechu*, glucosamine chondroitin, or placebo for 12 weeks. Discomfort, stiffness, and activities of daily living measured by the WOMAC questionnaire and VAS (pain/discomfort) were improved within all groups. Range of motion and distance walked were improved. There were no changes in TNF*α* levels for any of the products. There was a significant difference in urinary C-telopeptides of type II collagen (CTX-II), a marker of cartilage degradation between UP1306, and placebo after 12 weeks (*p* = 0.029). All efficacy measurements were improved from baseline to most time-points for UP1306, the comparator, and placebo without a significant association between the products. There was a significant difference between the changes of uCTX-II for UP1306 and placebo after 12 weeks. Early intervention with UP1306 aimed at reducing bone and cartilage degradation through reported inhibition of catabolic proinflammatory pathways may help to prevent joint cartilage damage. This study is registered with Clinical Trial ID ISRCTN15418623.

## 1. Introduction

Osteoarthritis (OA) is a chronic joint condition. It affects over 250 million people worldwide with significant impact on quality of life, health care, and society in general [1–3]. According to the World Health Organization (WHO) Global Burden of Disease Study 2010, hip and knee OA is the 11th leading cause of disability and shows a growing trend [4]. Vos et al. (2012) reported that knee OA accounts for 83% of the total burden of the disease [3]. There is no definitive cure or treatment to reverse the condition. Treatments are typically restricted to pain alleviation by a combination of pharmacological and nonpharmacological approaches. Unfortunately, only half of the patients experience pain reduction with pharmacological treatments [5]. In addition, modest improvements may be achieved by weight loss and physical activities [6]. Surgical interventions are sometimes warranted for severe cases. However, surgery is not always feasible due to constraints on costs or due to comorbidities [7, 8]. Therefore, there is a need for exploration of novel therapeutics for symptom management.

One such option is dietary supplementation with glucosamine and/or chondroitin. Despite their widespread use, the efficacy of glucosamine and chondroitin has undergone significant scrutiny over the past decade. The glucosamine/chondroitin Arthritis Intervention Trial (“GAIT Trial”) found no evidence for effective pain reduction in knee osteoarthritis; however, a subgroup analysis noted a statistical trend toward pain relief in patients with moderate to severe knee pain. According to Clegg et al., some of the discrepancies observed in relief of OA related discomfort or pain may be due to the varying dosages tested as well as other study design flaws [9]. Recent guidelines for the management of symptomatic knee osteoarthritis published by the Economic Aspects of Osteoporosis and Osteoarthritis ESCEO suggest use of prescription dose (1500 mg) of patented crystalline glucosamine sulfate as a first-line therapy [10]. Use of patented crystalline glucosamine sulfate has similar efficacy to nonsteroidal anti-inflammatory drugs (NSAIDs) and possibly better efficacy than that reported for paracetamol or acetaminophen preparations [11]. Chondroitin, either used in combination with glucosamine or alone, was also recommended as a first-line treatment [10].

Many medicines in use currently have been derived from natural occurring components of plants (botanicals and herbals). One example is UP1306 (*Acacia catechu* +* Morus alba*) composition. Preclinical studies showed a significant improvement in pain resistance and suppression of edema in rats and mice treated with UP1306 [12].

Acacia catechu (L.f.) Willd is from the Leguminosae family. It is a medium-size thorny deciduous tree, roughly 15 m tall. It is native to India and Burma and also found in China and parts of Bangladesh. Flavonoids, a type of water-soluble plant pigments, are the major class of compounds isolated from* Acacia* plants. Catechin is a major flavan in* Acacia* bark and heartwood, found primarily in green tea. Catechin has both antiviral and antioxidant activity. The anti-inflammation mechanism of Catechin is linked to its interaction with multiple targets involved in the inflammation process. Catechin modulates the inflammatory response by inhibiting the activity of cyclooxygenase-2 (COX-2) and lipoxygenase (5-LOX), lowering the activity level of platelet phospholipase A2, and significantly reducing platelet cyclooxygenase levels possibly by suppression of nuclear factor Kappa B (NF*κ*-B), inhibits the production of inflammatory cytokines tumor necrosis factor-alpha (TNF-*α*), interleukin- (IL-) 1, interleukin-2, interleukin-6, interleukin-8, and interleukin-12, and migration inhibitory protein through a number of mechanisms [13]. Extensive animal in vivo studies and human clinical trials compositions containing* Acacia* extract indicate that* Acacia* has great potential as a therapeutic agent for inflammatory diseases such as arthritis, irritable bowel syndrome, and inflammatory bowel syndrome [14–16]. Catechu black extract has been approved by the US FDA for food use as a natural flavoring substance and/or natural substance used in conjunction with flavors [17].

*Morus alba* L. (Moraceae), the mulberry or white berry plant, is native to northern China and has been cultivated and naturalized elsewhere from India and the Middle East to Southern Europe and recently to North America. In contemporary pharmacological research,* Morus alba* root bark has been reported to have antibacterial, antiviral, antioxidant, hypoglycemic, hypolipidemic, neuroprotective, antiulcer, analgesic, and anti-inflammatory properties. A variety of bioactive compounds from* Morus alba* root bark have shown in vivo and in vitro anti-inflammatory activity [18–20]. Based upon previous research, both prenylated flavonoids and stilbenoids in* Morus* ethanol extract contribute to joint protection and analgesic and anti-inflammatory properties [21, 22]. The combination of* Morus alba* root bark extracts with* Acacia catechu* heartwood extract in preclinical trials has demonstrated beneficial and synergistic effects with enhanced joint cartilage protection and anti-inflammatory and antinociceptive efficacy compared with either* Acacia catechu* heartwood or* Morus alba* root bark extracts alone by possibly acting upon different biological targets. The purpose of this study was to determine the effects of UP1306 on discomfort (onset and overall) and overall function when taken by individuals with OA of the knee for a 12-week period.

Safety was also assessed. Furthermore, the effects of 12-week use of UP1306 as compared to glucosamine chondroitin and placebo on inflammation and bone metabolism were measured.

## 2. Materials and Methods

This was a prospective, randomized, double-blind, comparator, and placebo-controlled parallel-group clinical trial. This study was approved by the Aspire Institutional Review Board (IRB) (Santee, CA) on February 14, 2014. The study was approved by the IRB as “UNI‐OA‐2014” and was submitted and registered with ISRCTN as ISRCTN15418623.

The subjects included 135 adults aged 35 to 75 years with a BMI < 35 kg/m^2^ who had knee pain for at least 15 of the 30 days prior to starting the study, had symptoms of knee pain for at least 6 months prior to starting the study, and had a Kellgren-Lawrence grade of I, II, or III according to the screening X-ray [23]. Qualified subjects (*n* = 135) were scheduled a second visit where they rated their knee discomfort/pain using a 10-point visual analog scale (VAS) (scores ≥ 6 inclusionary). Subjects with an inclusionary VAS-discomfort rating were randomized to receive UP1306 (*n* = 45), glucosamine and chondroitin combination (*n* = 45) or placebo (*n* = 45). The subjects were allocated, in equal probability, to the three product groups, using a Block 6 randomization scheme. Each group of six consecutively enrolled subjects were allocated among the three products (UP1306, the glucosamine chondroitin comparator and placebo), two subjects to each product, in random order. The study was double-blinded, so neither the subjects nor the study staff knew which product the subject was receiving. The glucosamine chondroitin combination was used as a positive control. This control was chosen because this treatment is a commonly used nutritional supplement [24], and UP1306 is potentially used by a similar demographic.

Study procedures included questionnaire completion Western Ontario and McMaster Universities Arthritis Index (WOMAC) used to assess pain, stiffness, and physical function in patients with hip and/or knee osteoarthritis, a six-minute walking test (6MWT), and a range of motion test using a goniometer. These procedures were performed at each of the six study visits so that the study products' effects on discomfort, physical function, range of motion, quality of life, and overall function could be determined.

Blood and urine were collected on days 0 and 84 to explore the effects of the study product on inflammation and bone metabolism. Blood was also used for assessing the safety of the product and a comprehensive metabolic panel, complete blood count with differential including platelets, and prothrombin time/international normalized ratio (PT/INR) were performed.

Throughout the duration of the study, subjects completed a Discomfort Diary consisting of a VAS. Subjects were asked to complete the diary daily for the first seven days and weekly for the remainder of the study. Subjects were also instructed to maintain their current diet and activity level throughout the study.

The subjects were provided with UP1306 (100 mg per capsule); active ingredients are standardized bioflavonoid extracts from root bark of* Morus alba* (white mulberry) and heartwood of* Acacia catechu* (*Senegalia catechu*) and inactive ingredients are Microcrystalline Cellulose, Magnesium Stearate (vegetable), and Silicon Dioxide. The no-observable-adverse effect-level (NOAEL) for UP1306 is 2000 mg/kg/day in a subchronic 90-day oral toxicity in vivo study (Data on file, Unigen Pharmaceuticals, Seattle, WA). Glucosamine (375 mg per capsule) and chondroitin (300 mg per capsule) combination: active ingredients are glucosamine hydrochloride and chondroitin sulfate; inactive ingredients are Magnesium Stearate (vegetable) and Silicon Dioxide. Placebo: inactive ingredients are Microcrystalline Cellulose, Magnesium Stearate (vegetable), and Silicon Dioxide. Subjects were instructed to take two capsules with a morning meal and two capsules with an evening meal (four capsules per day). Subjects were instructed to take the capsules with up to 8 ounces of water. Subjects had to take the capsules on 84 (±5 days) consecutive days.

After passing the screening visit and being entered into the study, subjects were provided with rescue medication (acetaminophen) and were asked to bring the unused rescue medication to each follow-up visit so that rescue medication usage could be determined. At day 0 visit, subjects were provided with the study product and were asked to bring the unused product to each follow-up visit so that compliance with product administration instructions could be determined (pill count method). Subjects were allowed to take up to 2 grams of acetaminophen daily in addition to their assigned study product (rescue medicine). However, they were not allowed to take the acetaminophen for the 2 days prior to each study visit. Subjects were provided acetaminophen at visits 1 through 6 and acetaminophen usage was calculated at visits 2 through 7. Rescue medication usage was determined by counting the number of tablets returned and subtracting that from the number provided and this was recorded as a % used. The amount of rescue medication taken was used as an indicator of the efficacy of the active product.

### 2.1. Efficacy Variables

The efficacy variables consisted of overall discomfort as measured by the WOMAC by evaluating the subscores for the domain of pain, VAS-discomfort ratings, VAS-discomfort weekly rating, and rescue medication use over 12 weeks. The acute effects were measured by VAS-discomfort ratings and rescue medication use over the first 7 days of product use. Overall function was measured by WOMAC pain, stiffness, and activities of daily living subscores, range of motion testing via goniometer testing, and distance walked during the Six-Minute Walk Test [25, 26]. Inflammation and bone metabolism were analyzed by blood levels of TNF*α* levels and urinary C-terminal crosslinking telopeptide of type II collagen (CTX-II).

### 2.2. Safety Variables

Safety variables consisted of blood work (comprehensive metabolic panel) (glucose, blood urea nitrogen (BUN), creatinine (Cr), aspartate aminotransferase (AST), alanine aminotransferase (ALT), alkaline phosphatase (ALP), bilirubin, total protein, albumin, calcium, chloride, carbon dioxide (CO2), sodium, potassium, and calculated estimated glomerular filtration rate (e-GFR)), complete blood count with differential (red blood cells (RBC), white blood cells (WBC), hemoglobin (Hgb), hematocrit (Hct), mean corpuscular volume (MCV), mean corpuscular hemoglobin (MCH), mean corpuscular hemoglobin concentration (MCHC), red blood cell distribution width (RDW), platelets, mean platelet volume (MPV), and prothrombin time/international normalized ratio (PT/INR)), blood pressure, heart rate, adverse events, and subjective remarks.

### 2.3. Examination of Data and Descriptive Statistics

All acquired variables were summarized by time point and by product. Numerical variables were presented as mean, standard deviation, count, median, and range (minimum to maximum value). Changes from baseline were summarized and presented in the same way and also included a *p* value indicating the significance of the change from baseline within that product group. Graphically displayed changes from baseline are displayed as plots of mean value versus time. The graphs contain vertical error bars around the mean values, indicating standard errors of the mean. Values and changes from baseline were compared between groups (UP1306, glucosamine and chondroitin combination, and placebo).

Excel 2010 (Microsoft Corp, Redmond WA) was used for data entry, validation, and restructuring. All descriptive statistics, graphs, parametric and nonparametric comparison of means, and Fisher's exact tests were generated using the “R” statistical/graphical programming system, ver.3.0.1 (R Foundation for Statistical Computing, https://www.r-project.org/).

## 3. Results

### 3.1. Demographics and Compliance

The baseline characteristics of the subjects are summarized in Table 1. All baseline demographic characteristics were adequately balanced between products; this suggests that any changes in response to the treatment are not due to variability in group characteristics. Compliance was measured via the pill counting method. Compliance with the study product was excellent with overall product compliance for all three products greater than 95%.

### 3.2. Efficacy

#### 3.2.1. WOMAC-Pain Subscore

**Figure 1:**
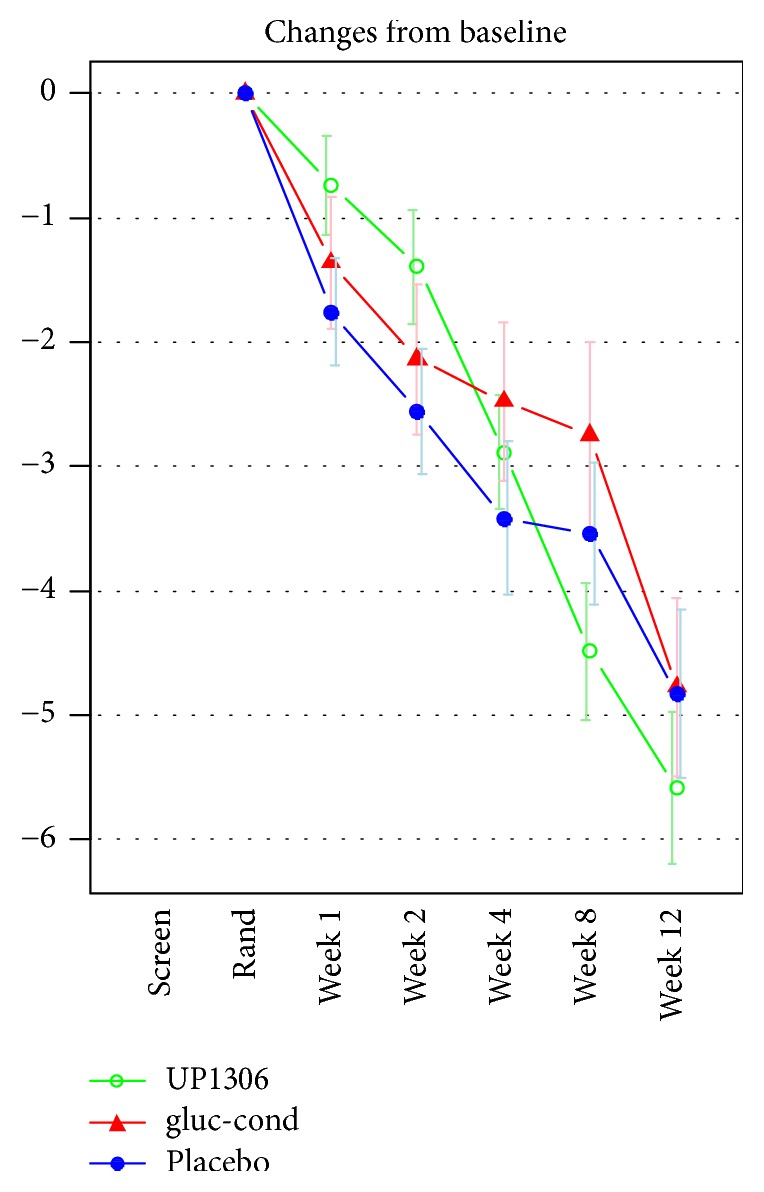
Changes in WOMAC-pain subscore from baseline to week 12. (i) *x*-axis = time-points/*y*-axis = change in WOMAC-pain scores.

For all three products, there were statistically significant within-group decreases in pain from baseline over the course of the study (Figure 1). The WOMAC-pain subscore was decreased by 51% (SD = 30) for UP1306, by 45% (SD = 41) for glucosamine chondroitin, and by 46% (SD = 40) for placebo. These decreases were not statistically significant between groups (*p* = 0.753). The only statistically significant difference between groups was the decrease from baseline to day 56 for UP1306 over the glucosamine chondroitin comparator (*p* = 0.048).

#### 3.2.2. WOMAC Stiffness Subscore

For all three products, there were statistically significant within-group decreases in stiffness from baseline over the course of the study. The WOMAC-pain stiffness was decreased by 45% (SD = 37) for UP1306, by 38% (SD = 45) for glucosamine chondroitin, and by 56% (SD = 37) for placebo. These decreases were not statistically significant, though, between groups (*p* = 0.115). The only statistically significant difference between groups was the decrease from baseline to day 14 for placebo over UP1306 (*p* = 0.050).

#### 3.2.3. WOMAC Activities of Daily Living

Although there were statistically significant within-group decreases in activity of daily living scores for all three products from baseline to all time-points, there were no statistically significant differences between the three products (*p* = 0.853).

#### 3.2.4. VAS-Discomfort Ratings

VAS-discomfort ratings for past 24 hours, first week, and past week are given in Table 2. There were statistically significant within-group decreases in past 24-hour discomfort ratings for all three products from baseline to all time-points, but there were no statistically significant differences between the three products (*p* = 0.935).

There were statistically significant within-group decreases in weekly discomfort ratings for all three products from baseline to all time-points, there were no statistically significant differences between the three products (*p* = 0.680). Although there were statistically significant within-group decreases in daily discomfort ratings for all three products from baseline to many time-points, there were no statistically significant differences between the three products (*p* = 0.811).

#### 3.2.5. Rescue Medication Use

There was an early trend for less rescue medication usage (calculated as a % used) by the UP1306 group as compared to placebo over the first month of the study (UP1306 −6.9%  ±  28.6 (43) versus −18.2%  ±  32.9 (41): *p* = 0.066. This greater usage of rescue medication by the placebo group as compared to the UP1306 group during the initial stages (first month) of intervention may have clinical relevance. There were no statistically significant differences in rescue medication use between the three products over the 12 weeks of study period (*p* = 0.812). The mean overall use was 18.8% (SD = 17.9) for UP1306, 19.0% (SD = 21.0) for glucosamine and chondroitin, and 21.2% (SD = 17.1) for placebo.

#### 3.2.6. Range of Motion

Range of motion results can be found in Table 3. For all three products, there were statistically significant within-group increases in extension scores (improved range of motion) from baseline over the course of the study. These increases were not statistically significant, though, between groups for (*p* = 0.763). The only statistically significant differences between groups were the increases from baseline to day 28 for the comparator and placebo over UP1306 (*p* = 0.018 and *p* = 0.042, resp.). Although there were statistically significant within-group decreases in flexion scores (improved range of motion) for all three products from baseline to many time-points, there were no statistically significant differences between the three products (*p* = 0.944).

#### 3.2.7. Distance Walked (6MWT)

There were statistically significant within-group increases in distance walked for all three products from baseline to all time-points. However, there were no statistically significant differences between the three products for the distance covered (*p* = 0.183; data not shown).

#### 3.2.8. Inflammation and Bone Metabolism

There were no statistically significant changes in TNF*α* levels within-group for any of the three products. There was also no statistically significant difference in the change from baseline to day 84 between groups (*p* = 0.786).

**Figure 2:**
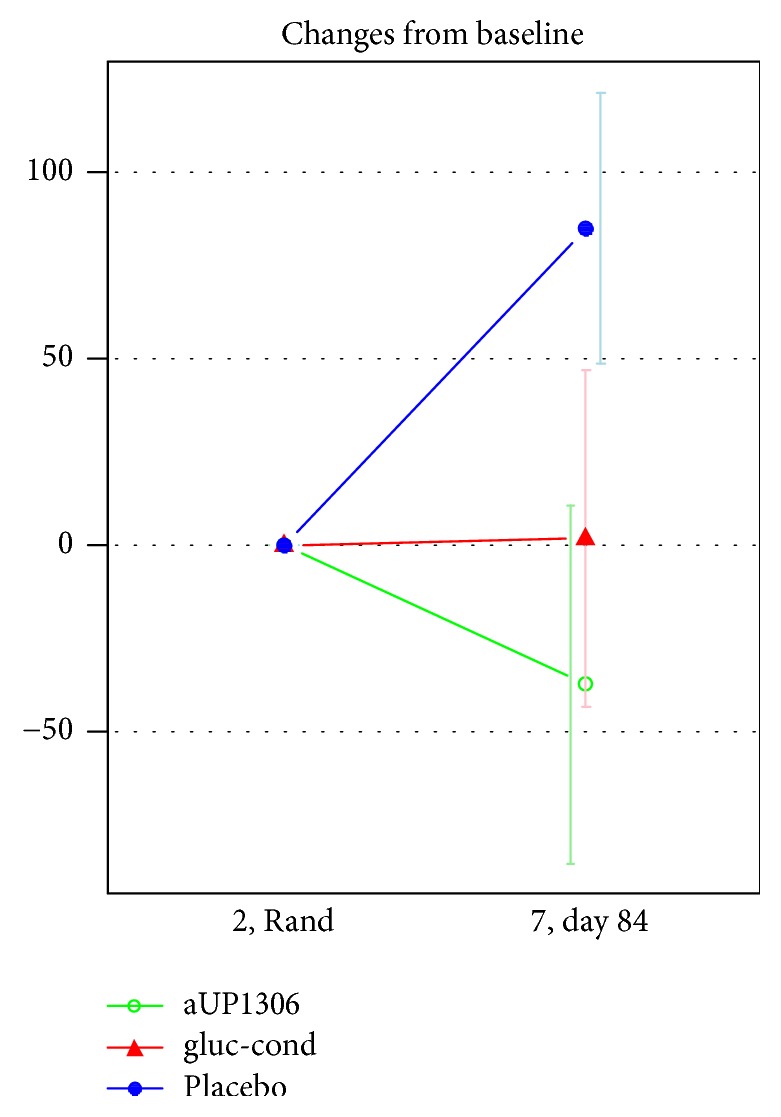
Changes in CTX-II from baseline to 12 weeks. (i) *x*-axis = time-points; *y*-axis = change in CTX-II levels.

There was a statistically significant difference in urinary CTX-II levels between the changes for UP1306 and placebo after 12-week product use (*p* = 0.029) (Figure 2).

### 3.3. Safety

#### 3.3.1. 12-Week Changes in Vital Signs

The results of the 12-week changes in vital signs are listed in Table 4. There were statistically significant increases in systolic and diastolic blood pressure within the UP1306 group (*p* = 0.010 and *p* = 0.036, resp.). This increase trended toward significance between products (*p* = 0.073 and *p* = 0.077, resp.). The changes from baseline in observed vital signs were not considered of clinical significance as both screening and baseline values were used (which did not always remain equal) and all values and tests remained within normal limits, resulting in these changes deemed not clinically significant nor meaningful.

#### 3.3.2. 12-Week Changes in Safety Lab Values

There were statistically significant changes for various laboratory markers within-group for all three products but none of these changes were of clinical significance. There was one between product difference of statistical significance for WBC (white blood cells) (*p* = 0.034) but this too was not of clinical significance. Values remained within normal limits and were considered not clinically meaningful.

#### 3.3.3. Adverse Events

No serious adverse events (AEs) were observed during the course of the study. A total of 43 adverse events were observed among 30 of the 133 subjects in the safety population. Fifteen (15) of the AEs were observed in the subjects in the UP1306 group, 10 AEs were observed in the glucosamine and chondroitin combination group, and 18 AEs were observed in the placebo group but none were determined to be clinically significant. Gastrointestinal disorders and infections and infestations were most frequently reported.

Fourteen (14) of the 43 total AEs, among seven subjects, were considered probably or possibly related to study product; the others were considered unlikely or definitely not related to study product. Among the 14 probably or possibly related AEs 3, AEs occurred among 2 subjects in the UP1306 group, 2 AEs occurred among 2 of the subjects in the glucosamine and chondroitin combination group, and 9 AEs occurred among 3 of the subjects in the placebo group.

There was no significant association between the test product and the frequency of occurrence of adverse events.

## 4. Discussion

In this trial, treatment with UP1306, glucosamine and chondroitin, and placebo during a 12-week period resulted in significant within-group improvements from baseline to most time-points for discomfort and overall function. There were no significant differences in frequency and occurrence of adverse events between the products indicating that UP1306 and glucosamine chondroitin have similar safety outcomes which is important considering the widespread use of GC and potentially UP1306.

The significant differences observed between products were as follows: the WOMAC-pain score decreased significantly from baseline to day 56 for UP1306 over the comparator (glucosamine and chondroitin combination) (*p* = 0.048). The WOMAC stiffness score decreased significantly from baseline to day 14 for placebo over UP1306 (*p* = 0.050). The range of motion as measured by extension improved significantly from baseline to day 28 for the comparator and placebo over UP1306 (*p* = 0.018 and *p* = 0.042, resp.). The UP1306 group took less rescue medication than the placebo group in the beginning, suggesting that there is an early effect for pain/discomfort relief with UP1306. The lack of superior benefit for range of motion when compared to the other test groups is not surprising since all had within-group improvements.

A significant difference between UP1306 and placebo after 12 weeks of product use was found for urine CTX-II. CTX-II is a marker for bone metabolism and is associated with the progression of osteoarthritis [27]. Type II collagen is a major structural component of cartilage tissue [28]. Measurements of fragments derived from this protein may represent a specific index for cartilage degradation associated with osteoarthritis [29]. The decreased amount of urinary CTX-II after treatment with UP1306 suggests that UP1306 might reduce the risk for the progression of osteoarthritis. This biological effect of UP1306 deserves further study as it appears to reduce the chances of osteoarthritis progression. It is interesting to note that while there were significant changes in CTX-II, this did not translate into consistent observed changes in the subjective validated scales regarding quality of life (WOMAC or VAS). The UP1306 group did experience significant improvement in WOMAC-pain score superior to the study comparator by day 56 (~2 months); this superiority did not remain by day 84 (week 12). This discrepancy very well may be a function of the sensitivity of the subjective questionnaires or that the positive bone effects take longer than 12 weeks to be physically felt as a function of activities of daily living and self-rated pain or discomfort levels. Thus the physiological significant changes observed without concomitant changes in the subjective scales used make this product worthy of more research that perhaps would demonstrate subjective benefit in durations longer than 12 weeks.

Bone is constantly undergoing remodeling. Cross-linked telopeptides collagens are the products in the remodeling process. While telopeptide of type I collagen accounts for about 90% of the organic matrix of bone, type II collagen is the major organic constituent of joint cartilage. Disruption of the structural integrity of cartilage is the major histological finding in osteoarthritis and rheumatoid arthritis. Following the degradation of cartilage, fragments of C-terminal cross-linked telopeptide type II collagen (CTX-II) are released into circulation and subsequently secreted into urine. Therefore, CTX-II is considered a viable biomarker for joint cartilage degradation and OA disease progression. In multiple studies, urinary CTX-II has been reported to be useful indicator in progression of osteoarthritis and early indication of rheumatoid arthritis [30, 31].

In this clinical trial, urinary levels of collagen type II C telopeptide fragments (uCTX-II) were measured by the CartiLaps ELISA assay. The concentration of the uCTX-II ELISA (mg/l) was standardized to the total urine creatinine (mmol/l): concentration/creatinine ng/mmol. uCTX-II biomarker values collected from the human clinical study were not evenly distributed and consequently the mean values and standard deviations were calculated by nonparametric statistics which may have influenced results. However, there was a statistically significant difference between the changes of uCTX-II for UP1306 and placebo groups after 12-week product use (*p* = 0.029).

Urinary CTX-II levels are useful for detecting populations at high risk of joint cartilage damage progression early in the disease. The elevated uCTX-II also suggests that these patients suffer increased bone/cartilage degradation, even in the absence of severe joint symptoms. Urinary CTX-II levels are correlated with joint space narrowing as well [26]. Early intervention with UP1306 aimed at reducing both bone and cartilage degradation through reported inhibition of catabolic proinflammatory pathways may help to prevent subsequent joint cartilage damage [5]. This association needs to be confirmed in other, larger and longer human clinical studies, in which these findings have important clinical implications for the management of osteoarthritis, rheumatoid arthritis, and other cartilage degradation related conditions.

## 5. Conclusions

In conclusion, all efficacy measurements were improved from baseline to most time-points for UP1306, the comparator, and placebo without a significant association between the products. There were no differences in the frequency and occurrence of adverse events. Interestingly enough, at early time-points in the study, those taking the UP1306 experienced significantly greater reduction in stiffness and pain as compared to the other groups as assessed from pain medication use. While not all efficacy markers were positive for superiority over the comparator or placebo, the fact that there were less stiffness and pain experienced, coupled with the biological marker for osteoarthritis progression being positively impacted only in the UP1306 group, leads us to believe that this product deserves further attention as it seems to have viability within the confines of this study. To further investigate, validate, and extend the findings, a next step would be to replicate this research in future studies, extending the study duration to determine translation of potential osteoarthritis relief and protection.

## Figures and Tables

**Table 1:** Baseline characteristics of subjects.

Product	UP1306	Glucosamine and chondroitin combination	Placebo	*p* value
*N* =	43	44	41	
Age years	60.4 ± 9.1 (43)	57.4 ± 8.1 (44)	57.1 ± 8.6 (41)	0.147
	61 (41–75)	57.5 (39–70)	59 (38–75)	
Gender				
Female	32 (74%)	29 (66%)	33 (80%)	0.318
Male	11 (26%)	15 (34%)	8 (20%)	
Total	43 (100%)	44 (100%)	41 (100%)	
Ethnicity				
Hispanic	42 (98%)	44 (100%)	40 (98%)	0.545
Non-Hispanic	1 (2%)	0 (0%)	1 (2%)	
Total	43 (100%)	44 (100%)	41 (100%)	
Race				
Afr_Amer	5 (12%)	4 (9%)	4 (10%)	0.936
Caucasian	38 (88%)	40 (91%)	37 (90%)	
Total	43 (100%)	44 (100%)	41 (100%)	
Height cm	161.9 ± 8.3 (43)	163.7 ± 8.5 (44)	161.7 ± 7.5 (41)	0.479
	160.1 (145–183)	164 (141–186)	162 (146.9–177.8)	
Weight kg	74.7 ± 13.7 (43)	78.6 ± 12.3 (44)	75.4 ± 11.9 (41)	0.315
	74.5 (46.6–106)	77.9 (51.7–108.2)	74.5 (53.2–97.1)	
BMI kg/m^2^	28.4 ± 3.8 (43)	29.3 ± 3.5 (44)	28.8 ± 4.0 (41)	0.527
	27.9 (20.4–34.8)	30.1 (21.6–34.9)	29.4 (20.9–34.9)	
Kellgren-Lawrence rating	2.14 ± 0.80 (43)	2.00 ± 0.89 (44)	1.83 ± 0.86 (41)	0.253
	2 (1–3)	2 (1–3)	2 (1–3)	

**Table 2:** VAS-discomfort ratings.

	UP1306	Glucosamine and chondroitin combination	Placebo	*p* value		
				All three products	UP1306 versus G&C	UP1306 versus placebo
24 hours						
Change from baseline to day 84	−3.47 ± 1.98 (43)	−3.32 ± 2.31 (44)	−3.49 ± 2.66 (41)	0.935	0.922	0.953
	−4 (−8–1)	−3 (−7–3)	−4 (−8–3)			
	*p* < 0.001^*∗*^	*p* < 0.001^*∗*^	*p* < 0.001^*∗*^			

First week						
change from baseline to day 6	−1.14 ± 1.87 (42)	−1.16 ± 1.52 (44)	−1.37 ± 1.85 (41)	0.811	0.879	0.610
	−1 (−5–3)	−1 (−5–1)	−1 (−6–2)			
	*p* < 0.001^*∗*^	*p* < 0.001^*∗*^	*p* < 0.001^*∗*^			

Past 7 days						
change from baseline to week 12	−3.49 ± 2.10 (43)	−3.09 ± 2.52 (44)	−3.49 ± 2.63 (41)	0.680	0.582	0.902
	−4 (−8–1)	−3 (−7–3)	−4 (−8–1)			
	*p* < 0.001^*∗*^	*p* < 0.001^*∗*^	*p* < 0.001^*∗*^			

^*∗*^ *p* < .05, the value is significant.

**Table 3:** Range of motion.

	UP1306	Glucosamine and chondroitin combination	Placebo	*p* value		
				All three products	UP1306 versus G&C	UP1306 versus placebo
Extension change from baseline to day 84	1.65 ± 2.43 (43)	1.93 ± 3.16 (44)	1.54 ± 1.89 (41)	0.763	0.445	0.683
	0 (−1–10)	0 (−2–14)	2 (−2–6)			
	*p* < 0.001^*∗*^	*p* < 0.001^*∗*^	*p* < 0.001^*∗*^			

Flexion change from baseline to day 84	−8.3 ± 7.1 (43)	−8.0 ± 8.2 (44)	−7.8 ± 8.2 (41)	0.944	0.867	0.930
	−8 (−25–8)	−6 (−25–4)	−6 (−32–7)			
	*p* < 0.001^*∗*^	*p* < 0.001^*∗*^	*p* < 0.001^*∗*^			

^*∗*^Significant: *p* ≤ 0.05.

**Table 4:** Change from baseline in vital signs over 12 weeks.

	UP1306	Glucosamine and chondroitin combination	Placebo	*p* value
Systolic BP mm Hg	5.5 ± 13.3 (43)	1.0 ± 16.2 (45)	−1.7 ± 14.0 (43)	0.073^#^
	5 (−25–34)	3 (−42–30)	−1 (−35–22)	
	*p* = 0.010^*∗*^	*p* = 0.688	*p* = 0.436	

Diastolic BP mm Hg	2.6 ± 7.9 (42)	1.6 ± 8.7 (45)	−1.2 ± 7.1 (43)	0.077^#^
	2.5 (−12–20)	3 (−28–18)	−1 (−20–17)	
	*p* = 0.036^*∗*^	*p* = 0.230	*p* = 0.288	

Heart rate beats/minute	−1.2 ± 7.3 (43)	1.3 ± 7.7 (45)	−1.3 ± 8.0 (43)	0.205
	−1 (−26–12)	1 (−15–18)	−1 (−18–14)	
	*p* = 0.304	*p* = 0.277	*p* = 0.273	

^*∗*^Significant: *p* ≤ 0.05;  ^#^trending toward significance: *p* < 0.10, but *p* > 0.05.
